# Hygiene and sanitation public health risks in illicit alcohol production and retail in Zambia

**DOI:** 10.3389/fepid.2025.1461874

**Published:** 2025-04-28

**Authors:** Musawa Mukupa, Cosmas Zyambo, Masauso Moses Phiri, Richard Zulu, Tulani Francis L. Matenga, Kumbulani Mabanti, Anna Hainze, Ahmed Ogwell, William DeJong, Dhally M. Menda, Angela Rizzo, Fastone Goma, Tom Achoki

**Affiliations:** ^1^ Centre for Primary Care Research, Lusaka, Zambia; ^2^ Department of Community and Family Medicine, School of Public Health, University of Zambia, Lusaka, Zambia; ^3^ Department of Pathology and Microbiology, School of Medicine, University of Zambia, Lusaka, Zambia; ^4^ Department of Health Promotion and Education, School of Public Health, University of Zambia, Lusaka, Zambia; ^5^ Brands on a Mission, Nairobi, Kenya; ^6^ United Nations Foundation, Washington, DC, United States; ^7^ Department of Public Health and Community Medicine, Tufts University, Medical School, Boston, MA, United States; ^8^ Churches Health Association of Zambia, Lusaka, Zambia; ^9^ AB InBev Foundation, New York, NY, United States; ^10^ African Institute for Health Policy, Nairobi, Kenya

**Keywords:** illicit alcohol, hygiene, sanitation, alcohol production, alcohol retailers

## Abstract

**Introduction:**

Illicit alcohol, existing outside legal frameworks, lacks safety standards and may contain harmful substances. In Africa, the illicit alcohol market is pervasive, contributing to significant public health challenges. The state in Zambia may not be so different where abuse of alcohol was associated to unintended injuries, suicidal ideation and physical fights that in some cases lead to death. This mirrors these challenges; according to the Zambia Stepwise Survey for Non-Communicable Diseases (NCDs) Risk Factors (2017), 21.7% of adults consume alcohol, with urban areas experiencing higher rates of illicit production.

**Aim:**

The study aimed to assess the public health risk implications associated with the production and retail of illicit alcohol based on the hygiene and sanitation of these premises. A quantitative approach was employed in Livingstone, Lusaka, and Ndola.

**Methods:**

We employed a quantitative approach was conducted in Livingstone, Lusaka, and Ndola. A hygiene and sanitation observation tool obtained from the Lusaka City Council was adapted and utilized to evaluate the conditions of these premises.

**Results:**

The study found that Livingstone exhibited the highest rate of unavailability of water supply at illicit alcohol production sites, with 18% lacking access to water, while Lusaka had 13% without water supply of the zones selected, all sites in Ndola had water supply.

**Conclusion:**

Given the growing trend of consuming illicit alcohol in such unsanitary environments, urgent interventions are warranted. The study recommends the implementation of enforcement of regulations, including regular inspections and enhanced enforcement mechanisms, to ensure hygienic and sanitary production practices.

## 1 Introduction

Alcohol consumption is a prevalent cultural practice, but its excessive use poses significant public health concerns globally with heavy episodic alcohol use affecting most adults and is the third leading cause of death (1–3). According to the WHO, in 2022, harmful use of alcohol accounted for more than 3 million deaths, making up 5.3% of all deaths globally (4). In Africa, the illicit alcohol market is pervasive, contributing to significant public health challenges. A study conducted in Nigeria revealed a significant disparity between producers' knowledge and their actual hygiene practices, with a majority displaying poor practice levels (5)*.* The state in Zambia may not be so different where abuse of alcohol was associated to unintended injuries, suicidal ideation and physical fights that in some cases lead to death (6). This mirrors these challenges; according to the Zambia Stepwise Survey for Non-Communicable Diseases (NCDs) Risk Factors (2017), 21.7% of adults consume alcohol (7, 8), with urban areas experiencing higher rates of illicit production. The burden of age-standardized alcohol-attributable disease and injury was highest in the WHO African region, of which Zambia is a part (9).

The Zambia Stepwise Survey for Non-Communicable Diseases (NCDs) Risk Factors (2017) found that 21.7% of respondents were current alcohol drinkers, with significant gender differences (32.0% for men compared to 11.8% for women) (7). These may be prone to suicidal ideation, involved in fights and also be involved in road accidents (6, 10). The prevalence of current drinkers was highest among men aged 30–44 years (37.8%) and women aged 60–69 years (15.3%) (7). Urban women reported a significantly higher percentage of current drinkers (15.7%) than rural women (7.8%) (7). Additionally, the Zambia Global School Health Survey 2004 revealed that 42.6% of students in grades 7–10 across 47 schools in 9 provinces had consumed alcohol on one or more occasions in the last 30 days (11). This statistic poses a significant risk to children, as early exposure to alcohol can lead to a range of adverse health outcomes and behavioral issues. The consumption of alcohol at such a young age is associated with an increased likelihood of developing alcohol use disorders later in life, as well as heightened vulnerability to engaging in risky behaviors, including unprotected sex and substance abuse (12–14).

Safety and hygiene are crucial in alcohol production. The alcohol industry ideally adheres to Good Manufacturing Practice (GMP) standards. However, observations at illicit production sites reveal non-compliance with GMP guidelines (15). Illicit alcohol production typically occurs in informal settings characterized by low-income families, where hygiene and sanitation standards are often substandard or nonexistent (16–18). Common practices include the use of contaminated water sources, inadequate cleaning of equipment, and improper storage of raw materials. These practices create an environment conducive to microbial contamination, including bacteria, fungi, and molds, which can proliferate throughout the production process and contaminate the final product (18, 19).

The Zambia cholera outbreak of the 2023/24 rainy season showed Lusaka to be a major cholera hotspot and prompted recommendations to strengthen interventions such as area-targeted interventions (CATI). A report authored by the Africa Centres for Disease Control and Prevention (Africa CDC) further suggested improving water, sanitation, and hygiene interventions, recommending quality monitoring, disinfection, and inspections. The Africa CDC press release also confirmed that the Zambian cholera outbreak in October 2023 was first detected in Matero and Kanyama, areas known for illicit alcohol production and retail, although no studies have directly linked the outbreak with these activities (20).

The existing body of research on sanitation and hygiene often overlooks the critical intersection between public health and illicit alcohol production and retail. This knowledge gap represents a significant deficiency in understanding the public health risks associated with this industry. By bridging this divide, our study aims to illuminate the relationship between sanitation, hygiene, and public health risks in illicit alcohol production and retail. Through comprehensive investigation and analysis, this paper seeks to provide crucial insights to inform targeted interventions, policies, and public health strategies to mitigate adverse effects on individuals and communities. Addressing this research gap is imperative to safeguard public health and promote sustainable development in the context of illicit alcohol production and retail.

This study aims to bridge the gap between global knowledge and the realities in Zambia by examining hygiene and sanitation practices in illicit alcohol production in Zambia. The results from this study will inform targeted interventions that can mitigate public health risks associated with this pervasive issue.

## 2 Methodology

### 2.1 Study design and sites

The study employed a quantitative approach and was conducted in three urban and peri-urban areas in Zambia: Livingstone, Lusaka, and Ndola. The study was conducted between June and December, 2023. The selection of these study sites was purposeful. Lusaka, the capital city of Zambia, and Ndola located in the Copper Belt region, were chosen due to their reported high consumption of illicit alcohol, attributed to undeclared (tax leakage) production of alcohol and smuggling. Livingstone was also purposefully sampled as a border city that shares a border with Namibia, Botswana, and Zimbabwe. Despite the choice of sites being justified based on the known high-risk areas for illicit alcohol production and consumption in Zambia, the potential limitation is the focus on urban and peri-urban areas. This may lead to the findings not being generalized to rural settings where alcohol production and consumption dynamics might differ.

### 2.2 Study setting

In Lusaka, we selected the following Zones or areas: COMESA market, Matero, Kanyama, and Mtendere. Kamanga, Bauleni, and Chibolya were purposefully chosen due to their reputation as hotspots for home-produced alcohols used for commercial purposes. In Ndola, we selected the main market areas: Main Masala, Chifubu, and Kaloko. Lastly, in Livingstone, we selected Town Centre (Zimbabwe market), Maramba, Linda, Libuyu, and Dambwa. We included all retailers within a 1 km radius who willingly consented to participate in the study. The data collection phase was time-bound and limited to one month.

### 2.3 Data collection

This study aimed to assess public health risk implications based on factors such as hygiene and sanitation in the production and retail of illicit alcohol. This was done by observation of the environment or surroundings of the place where the illicit alcohol is being distilled or sold. A hygiene and sanitation tool were adapted from the local city council to measure and ascertain levels of hygiene. The variables included presence of iron drums, solid waste management, water availability, toilets, and infestations. These variables were adapted from the local council tool because they are defined as key indicators of environmental health and sanitation by the local council, which directly impact the safety and quality of alcohol production. For example, the use of iron drums, especially if rusted or previously used for hazardous materials, poses a significant risk of chemical contamination i.e iron poisoning. Similarly, the availability of clean water and proper waste management reduces the likelihood of biological contamination, while the presence of toilets and pest infestations reflects the overall hygiene standards of the production environment, all of which are critical to public health risk.

The target study sites were places where illicit alcohol was produced and sold and where bulk opaque beers were served in barrels in the selected areas of Livingstone, Lusaka, and Ndola. The type of drums, the presence of water supply, and toilets were assessed using the checklist. Within the time-bound one-month limit of data collection fieldwork, 207 producers were recruited for the study.

We used experienced research assistants whom we trained for a period of four days, with the data collection tools pre-tested and refined on Day 5. Before fieldwork, a pre-study exercise of mapping the health zones in each city was undertaken to determine the number of alcohol producers and retailers to be reached in the main study. Data were primarily collected utilizing phone tablets with Open Data Collect (ODK). The socio-demographic collected included age, gender, education and marital status.

### 2.4 Statistical analysis

Statistical analyses were performed using STATA version 17. Descriptive statistics were calculated for all variables, including means, standard deviations, and frequencies. To evaluate hygiene standards among producers and retailers, we employed the chi-squared test to determine statistically significant differences in hygiene scores across different cities. A significance level of *p* < 0.05 was established, indicating that results with *p*-values below this threshold were considered statistically significant.

### 2.5 Data management

Data on hygiene and sanitation conditions were collected through structured questionnaires administered to the owners or managers of illicit alcohol premises. The questionnaire captured information on the cleanliness of production areas, availability of sanitation facilities, and safety measures to prevent contamination. Data were collected in Livingstone, Lusaka, and Ndola, and entered into statistical software following rigorous quality control procedures to ensure accuracy.

### 2.6 Ethical consideration

All potential participants were informed about the study and provided written consent before data collection. Data collection procedures were designed to avoid disrupting the normal flow of business. Interviewers were instructed to proceed with the observations only when the retailer/producer was not actively dealing with customers or in their immediate vicinity. Ethical approval for the study was obtained from the University of Zambia Biomedical Research Ethics Committee (UNZABREC REF. No. 4272-2023). Permission to conduct the study was sought from the National Health Research Authority (NHRA).

## 3 Results

### 3.1 Sociodemographic characteristics of participants

Based on Table 1, the participants were predominantly female across all three geographical areas (Livingstone, Lusaka, and Ndola). The female representation ranged from 84% to 93%, while males comprised 6% to 16%. The producers and retailers spanned various age groups. The largest group, at 20% to 38%, fell within the 36–45-year-old range, followed by 46–59-year-olds at 30% to 41%. 18–25year-olds constituted the smallest age group, ranging from 7% to 14%. In terms of education, a significant portion (40% to 56%) of the participants had primary school education (G1-G7). Those with no formal schooling ranged from 6% to 17%. Higher education levels were less common, with college/university degrees ranging from 3% to 4% and high school education (G10-12) at 7% to 23%. Regarding marital status, most producers and retailers (45%) were married, with city-specific variations ranging from 39% to 48%. The second largest group consisted of widowed individuals, representing 24% in Ndola, 26% in Lusaka, and 33% in Livingstone. As expected, most participants (ranging from 90% to 100%, with an average of 95%) were self-employed.

**Table 1:** Sociodemographic characteristics of retailers and producers.

Variable	Brewers/district (%)			Total (%)
	Livingstone (*n* = 68)	Lusaka (*n* = 121)	Ndola (*n* = 29)	
Sex				
Female	84	86	93	86
Male	16	14	7	14
Age				
18–25	7	9	14	9
26–35	16	14	14	14
36–45	20	28	7	23
46–59	38	30	41	34
≥60	19	20	24	20
Education				
No formal schooling	6	13	17	11
Primary school (G1-G7)	39	56	52	50
Secondary school (G8-G9)	28	18	21	21
High school (G10-12)	23	10	7	14
College University	4	3	3	4
Marital status				
Never married	9	10	10	10
Currently married	39	48	45	45
Separated	9	2	0	4
Divorced	10	14	21	14
Widowed	33	26	24	28
Employment status				
Government employee	0	0	0	0
Non-government employee	4	1	0	2
Self-employed	90	97	100	95
Non-paid/volunteer	1	0	0	0
Student	1	0	0	0
Homemaker	1	0	0	0
Retired	0	1	0	0
Unemployed (able to work)	1	2	0	1

**Table 2:** Proportion of illicit alcohol production sites and retailers by district and hygiene and sanitation indicators.

Indicator	Livingstone *N* = 67, %	Lusaka *N* = 112, %	Ndola *N* = 28, %	*P*-value
Solid waste	37.0	57.4	5.6	0.131
Toilet	46.7	53.3	0.0	0.201
Water supply	46.3	53.9	0.00	0.056
Infestation	47.9	38.8	13.2	0.000
Dirty drum	44.8	44.8	10.4	0.000
Iron storage	12.9	72.7	14.4	0.000
Plastic drums	27.5	58.2	14.3	0.000
Other materials	16.5	76.9	6.6	0.000

All indicators were statistically significant at (*p* < 0.05, chi2) except for solid waste, toilet and water supply.

### 3.2 Hygiene and sanitation of the premises

**Figure 1:**
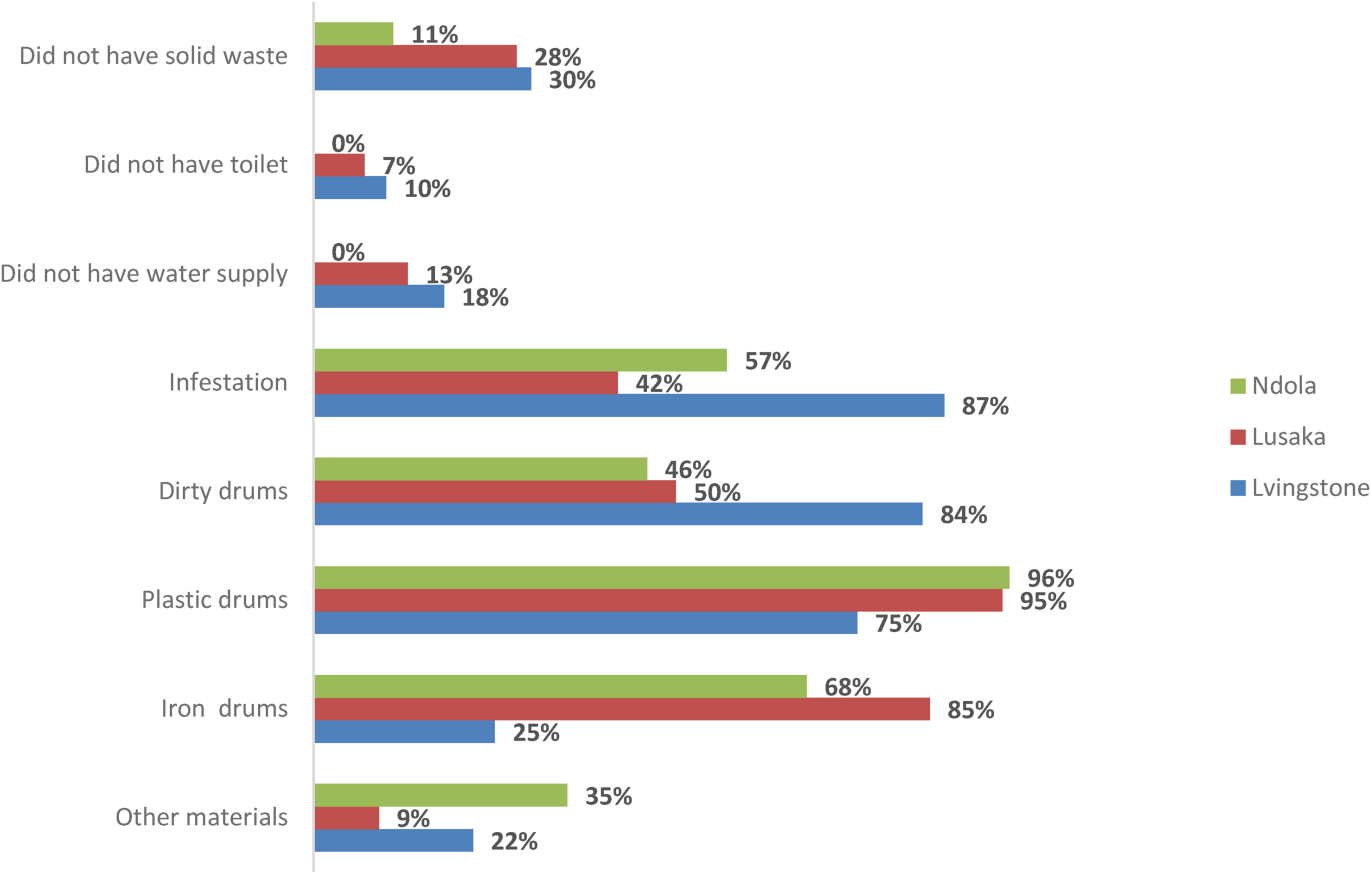
Hygiene and sanitation of the producers in production of illicit alcohol.

The data from 207 producers across Livingstone, Lusaka, and Ndola indicates significant public health risks associated with the production of illicit alcohol (Figure 1). Some key findings focus on improper storage containers: a high proportion (88%) of producers rely on plastic drums for storage, which can be susceptible to leaching chemicals or harboring bacteria if not properly cleaned and maintained. There was also the presence of dirty equipment, with more than half (60%) of producers found using dirty drums, further increasing the risk of contamination in the end product. We also found limited sanitation facilities, where a significant number of producers (68% in Luska, 10% in Livingstone) lack toilets, raising concerns about overall hygiene practices and potential fecal contamination. Solid waste mismanagement is evident, as only 26% of the production sites have proper management of solid waste, suggesting potential breeding grounds for pests and pathogens. While not the most prevalent concern, 13% of producers reported inadequate water supply, which could hinder proper cleaning and sanitation during production. A concerningly high proportion (58%) of producers reported infestations, which could contaminate ingredients or equipment and pose health risks to consumers.

These findings highlight the potential public health hazards associated with consuming illicit alcohol in Zambia. The use of improper containers, dirty equipment, and a lack of sanitation facilities significantly increases the risk of contamination by bacteria, parasites, or other harmful pathogens. Additionally, inadequate water supply and infestations further exacerbate these risks.

### 3.3 Comparison between districts

Illicit alcohol producers in Livingstone reported the highest percentages for deficiencies in solid waste management (30%), inadequate toilet facilities (10%) and water supply challenges (18%). Notably, the use of iron and dirty drums, were most prevalent among producers in Lusaka. Ndola and Livingstone, however, exhibited a higher proportion using storage materials other than plastic or iron drums. These findings highlight the critical public health risks associated with hygiene and sanitation in the illicit production of spirits and opaque alcohol across all three sites.

### 3.4 Comparative analysis across hygiene indicators

A comparative analysis of Livingstone, Lusaka, and Ndola across various hygiene indicators reveals distinct disparities (Table 2). Sites in Lusaka consistently displayed higher proportions across several indicators: solid waste management (57.4%), toilet facilities (53.3%), water supply (53.8%), iron storage (72.7%), presence of plastic drums (58.2%), and presence of other materials (76.8%). Livingstone generally had production sites and retailers with moderate proportions across indicators, such as solid waste management (37.0%), toilet facilities (46.6%), and water supply (46.1%). Ndola consistently reported lower proportions across several key indicators, including toilet facilities (0%), water supply (0%), and infestation (13.2%).

## 4 Discussion

The widespread proliferation of illicit alcohol production and retail sites across the country is a matter of great concern. Our investigation into the hygiene and sanitation of illicit production sites uncovers a complex landscape of unhealthy practices. The study showed a higher dominance of women in illicit alcohol production (Livingstone: F: 84%, M: 16%, Lusaka: F: 86%, M: 14%, Ndola: 93%, M: 7%). Ndola showed the highest percentage of women involved in the illicit trade. This may be associated with the presence of mines on the Copperbelt, which traditionally employ more men than women (21, 22). Currently, training institutions for mine workers, such as universities, colleges and technical institutes, are still dominated by male students, leading women to take up illicit alcohol production or trade as an alternative income-generation strategy (21, 23). The Zambian labor market shows similar trends, where women are more likely to be employed in the informal sector, such as retail, and where the median age of the population is relatively young (24, 25). The predominance of women in this sector reflects broader socio-economic trends where women often resort to informal sectors for income generation (21, 23).

The study showed that the age group most involved in the production and trade of illicit alcohol is between 46 and 59 years and most producers have formal education corresponding to primary school (G1-G7), are married (45%), and self-employed (81%). This could suggest that illicit alcohol serves as a source of income for some Zambian families. The younger group of producers and retailers, between the ages of 18 and 25 years, shows that Livingstone had 7%, Lusaka 9%, and Ndola had the highest with 14%. This young population is also at risk of drinking the illicit alcohol they produce or retail. This is because the illicit alcohol is cheaper and more accessible than licit or legally produced alcohol beverages sold at liquor stores (26, 27).

The study observed the sanitation and levels of hygiene in the production and retail sites. The variables observed included solid waste, toilets, water supply, infestations, dirty drums, iron storage, and plastic drums used for storage. The *P*- Value for infestations, dirty drums, iron storage, and plastic storages was significant. Ndola showed the lowest rate in the absence of water at the production sites.

The study revealed that Livingstone exhibited the highest unavailability of water supply at illicit alcohol production sites, with 18% lacking access to water of the 3 zones selected, while Lusaka had 13% without water supply of the 7 zones selected, and all sites in Ndola had adequate water supply from the 3 zones selected. These low rates of water availability could contribute to the spread of infectious diseases, such as recent outbreaks of cholera, as water and sanitation play a crucial role in cholera transmission. The unavailability of water supply at these sites could be as a result of high cost of either drilling boreholes or connecting to the public water supply system (28). The high incidence of cholera cases in Lusaka district, identifying it as a hotspot, can be attributed to localized outbreaks affecting specific wards or compounds, such as Mandevu, Matero, and Kabanana, which are often sites of illicit alcohol production (29). Zambia has recently experienced various cholera outbreaks, with the most recent occurring around October 2023 and continuing into 2024. This outbreak was highly fatal, with a case fatality rate of 4%, which is four times the World Health Organization threshold. Most of the cases occurred in December 2023 and January 2024, with Lusaka having more than 7,783 cases as reported by the Zambia National Public Health Institute (30, 31).

The widespread utilization of plastic drums, dirty containers, and inadequate toilet facilities poses significant public health and environmental risks. Moreover, solid waste mismanagement, insufficient water supply, and infestations exacerbate these concerns. The use of plastic materials is linked to various severe adverse effects such as cancers, birth defects, and impaired immunity (32). Regional disparities in hygiene and sanitation practices were also evident, with respondents in Lusaka reporting the highest percentages of deficiencies in solid waste management, inadequate toilet facilities, water supply challenges, and reliance on plastic drums. This could potentially lead to infestations with rodents and cockroaches, while respondents in Livingstone reported a higher prevalence of using iron and dirty drums. In contrast, Ndola exhibited a high proportion of using storage materials other than plastic or iron drums.

These findings underscore the critical need for stricter regulations and enforcement to ensure that all alcohol producers, including those currently producing and selling illicit alcohol, follow best practice regarding hygiene and sanitation. Public health education campaigns are essential to raise awareness of the risks associated with consuming illicit alcohol.

## 5 Conclusion and recommendation

Our investigation into hygiene and sanitation public health risks associated with illicit alcohol production and retail in Zambia highlights pressing concerns that demand immediate attention. To address these challenges effectively, we propose a multifaceted approach encompassing regulatory, educational, and supportive measures.

Comprehensive public awareness campaigns are vital to inform consumers about the health risks associated with consuming illicit alcohol. By enhancing public understanding, individuals can make informed decisions regarding their alcohol consumption habits, thereby reducing the incidence of related health issues. Moreover, stakeholders must collaborate to develop and promote alternative livelihoods for those engaged in illicit alcohol production, addressing the underlying socioeconomic factors driving participation in this illicit trade. Additionally, targeted training programs are essential for educating illicit alcohol producers on proper hygiene practices and the role they play in preventing disease outbreaks, such as cholera.

In conclusion, it is imperative that stakeholders including policymakers, community leaders, and health organizations—collaborate to establish comprehensive strategies aimed at mitigating these public health risks. By prioritizing interventions that address hygiene and sanitation in illicit alcohol production, we can protect vulnerable populations and promote healthier communities.

## Data Availability

The original contributions presented in the study are included in the article/Supplementary Material, further inquiries can be directed to the corresponding author.
